# A Two-Photon Microimaging-Microdevice System for Four-Dimensional Imaging of Local Drug Delivery in Tissues

**DOI:** 10.3390/ijms222111752

**Published:** 2021-10-29

**Authors:** Guigen Liu, Veronica Valvo, Sebastian W. Ahn, Devon Thompson, Kyle Deans, Jeon Woong Kang, Sharath Bhagavatula, Christine Dominas, Oliver Jonas

**Affiliations:** 1 Department of Radiology, Brigham and Women’s Hospital, Harvard Medical School, 221 Longwood Ave, Boston, MA 02115, USA; gliu19@bwh.harvard.edu (G.L.); valvo.veronica@gmail.com (V.V.); wahn1@bwh.harvard.edu (S.W.A.); devon.thompson96@gmail.com (D.T.); kyle_deans@dfci.harvard.edu (K.D.); sbhagavatula@bwh.harvard.edu (S.B.); cdominas@bwh.harvard.edu (C.D.); 2 Laser Biomedical Research Center, G. R. Harrison Spectroscopy Laboratory, Massachusetts Institute of Technology, Cambridge, MA 02139, USA; jwkang76@mit.edu

**Keywords:** biomedical microdevice, two-photon micro-endoscopy, optical sectioning, local drug delivery, tumors, in vivo testing

## Abstract

Advances in the intratumor measurement of drug responses have included a pioneering biomedical microdevice for high throughput drug screening in vivo, which was further advanced by integrating a graded-index lens based two-dimensional fluorescence micro-endoscope to monitor tissue responses in situ across time. While the previous system provided a bulk measurement of both drug delivery and tissue response from a given region of the tumor, it was incapable of visualizing drug distribution and tissue responses in a three-dimensional (3D) way, thus missing the critical relationship between drug concentration and effect. Here we demonstrate a next-generation system that couples multiplexed intratumor drug release with continuous 3D spatial imaging of the tumor microenvironment via the integration of a miniaturized two-photon micro-endoscope. This enables optical sectioning within the live tissue microenvironment to effectively profile the entire tumor region adjacent to the microdevice across time. Using this novel microimaging-microdevice (MI-MD) system, we successfully demonstrated the four-dimensional imaging (3 spatial dimensions plus time) of local drug delivery in tissue phantom and tumors. Future studies include the use of the MI-MD system for monitoring of localized intra-tissue drug release and concurrent measurement of tissue responses in live organisms, with applications to study drug resistance due to nonuniform drug distribution in tumors, or immune cell responses to anti-cancer agents.

## 1. Introduction

Recently, a biomedical microdevice [1] has emerged as a promising tool for high-throughput screening of drugs in vivo for the treatment of complex diseases, such as cancers. One of the key advances provided by this technology is the parallel efficacy assessment of up to 20 drugs loaded on the microdevice, which is implanted in situ in the diseased tissue and thus delivers results collected from the intact microenvironment. While the drugs release and interact locally with the tissue in its native state, a major limitation is that assessment of the tissue response still relies on removing the tissue from the body using a conventional biopsy at a single time point for in situ analysis [2,3,4]. Although this single endpoint examination method provides valuable static data, it misses key time-dependent phenomena such as the recruitment of immune and stromal cells which may play a key role in treatment resistance. In addition, different drugs may have time-dependent effects that are not captured with a single timepoint, which may lead to suboptimal evaluation of the tissue response.

To augment the potential of intratarget microdose testing, a microimaging system that functions as an in vivo “histology lab” should be integrated to better capture time-dependent effects and monitor tissue response continuously over time. To this end, we previously demonstrated a “lab-in-a-tumor” implantable microdevice (LIT-IMD) platform for multiplexed drug response testing in live tumors [5]. The LIT-IMD system included a commercial two-dimensional (2D) two-color fluorescence micro-endoscope with a customized graded index (GRIN) lens probe. Using this platform, we have successfully verified the feasibility of parallel imaging of drug diffusion and concurrent monitoring of cell death via two color channels. While the microimaging system can visualize the tissue response in a 2D manner, it integrates the fluorescence signals accumulated over the penetration depth of around 300 µm, and thus lacks three-dimensional (3D) imaging capabilities that are critical to the understanding of the drug-tissue interactions. Studies have shown that uneven drug exposure may significantly contribute to the resistance of cancers to chemotherapy [6,7,8,9]. Measurement of the drug penetration, distribution, and efficacy relies on advanced 3D microscopy techniques. A variety of 3D imaging methodologies have been adopted in biomedical research, such as confocal fluorescence microscopy for the imaging of *E. coli* nucleoid organization and dynamics [10] and transdermal delivery of avanafil [11], magnetic resonance imaging for multiphase steady-state imaging in pediatric congenital heart disease [12], X-ray computed tomography for the identification of mechanical stress distribution in suture and tendon applications [13] and the imaging of asthmatic human lungs [14], and near infrared spectroscopy for the investigation of glimepiride liposomal films [15]. These techniques are, however, not capable of directly measuring the heterogeneous intratumor drug concentration because of the limited penetration depth or insufficient resolution. Thus, there is a growing need for the development of a 3D microimaging-microdevice (MI-MD) system that is capable of investigating intratumor drug exposure heterogeneity. In addition, visualization of how immune cells respond to drugs is of great interest for developing combination treatments [16,17], and this advance would also necessitate 3D resolution.

To address the above-mentioned unmet need, in this work we develop and demonstrate such a system using GRIN lens based two-photon micro-endoscopy for four-dimensional (4D) drug penetration testing (three spatial dimensions plus the fourth dimension of time). Although similar GRIN lens based confocal or multiphoton micro-endoscopy has been investigated [18,19,20,21,22,23,24,25], this is the first instance in which such a 3D imaging system is integrated with multiplexed drug delivery into tumors, representing an important advance toward realizing the simultaneous measurement of drug distribution and tissue effect without the need for tissue removal by biopsy.

## 2. Results

### 2.1. Two-Photon MI-MD System

#### 2.1.1. System Configuration

**Figure 1:**
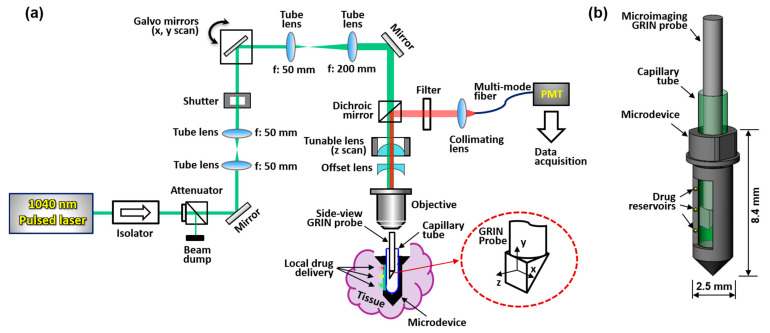
Two-photon MI-MD system for local drug delivery testing. (**a**) Schematic of the whole system, (**b**) close-up view of the MI-MD assembly. PMT, photomultiplier tube.

A schematic of the MI-MD system is shown in Figure 1a. A 1040 nm pulsed laser serves as the two-photon excitation source. Two galvo mirrors provide scans along x and y directions, and an electronically tunable lens (ETL) provides scanning along the z direction; the inset in Figure 1a shows schematically the x, y, and z scan directions. The z scan is implemented by tuning the convergence or divergence of the incident laser beam, which is controlled by the ETL in conjunction with an offset lens. ETLs have been shown to provide reliable and fast scan of focus in the z direction without the need for mechanical motion [26,27]. The ETL used in our experiments has a response time of < 2.5 ms. The microdevice is implanted in the tissue during testing and the side-viewing microimaging GRIN probe is inserted into the microdevice through a thin capillary tube which is optically clear and provides mechanical protection. The excited fluorescence signal is epi-reflected and sent to a photomultiplier tube (PMT) through a multimode fiber for image acquisition. The GRIN probe has a doublet configuration and delivers side-viewing imaging through a right-angle prism [20,22]. A more detailed description of the system setup and its components is provided in Section 4.1. A close-up view of the MI-MD assembly is shown in Figure 1b. The microdevices used throughout this work have an outer diameter of ~2.5 mm and a total length of ~8.4 mm, which accommodates the 1-mm-diameter GRIN probe with a larger field of view (FOV). An opening on one side of the device is created as the side-viewing window. Drug reservoirs are machined on both sides of the opening, and drugs are loaded in each of the reservoirs. The location and density of the reservoirs may vary for different experiments. At a desired time point following device insertion into tissue, center of the prism is aligned with a drug reservoir while the drug is released locally into the tissue. After a 3D image has been acquired, the probe is translated to another reservoir location for next image acquisition.

#### 2.1.2. FOV and Resolution

**Figure 2:**
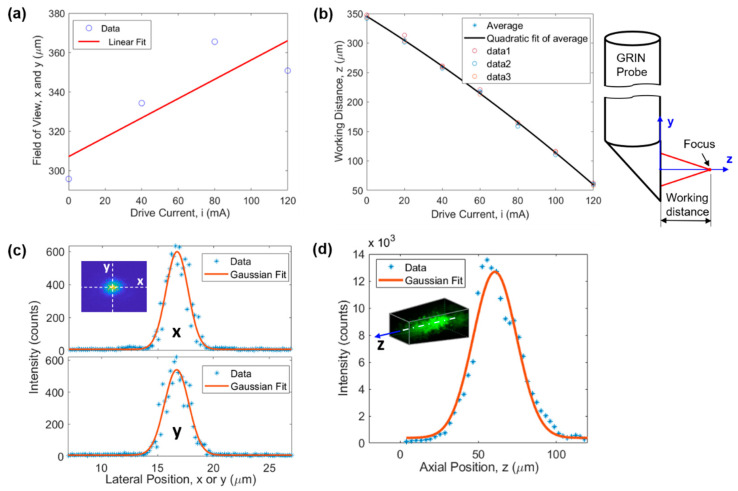
Characterization of the microimaging system. (**a**) FOV and (**b**) WD as a function of the drive current of the ETL, (**c**) lateral (i.e., x and y directions) and (**d**) axial (i.e., z direction) resolution.

**Figure 3:**
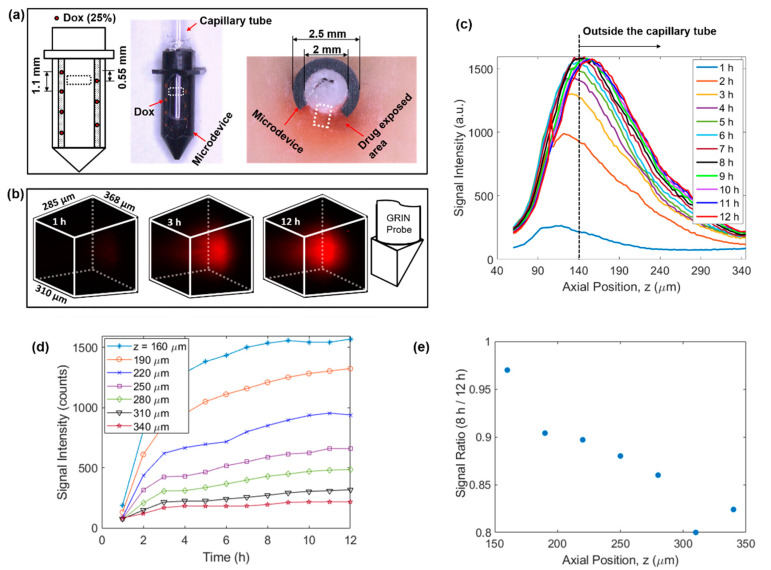
4D imaging of local doxorubicin delivery in breast tissue phantom using the developed MI-MD system. (**a**) Schematic (left) and image of the drug-loaded microdevice (middle) and cross-sectional view around the reservoir level after the testing (right). The region for imaging is designated roughly by the dashed squares. (**b**) Temporal evolution of the drug diffusion process captured by the MI-MD system. Orientation of the imaging probe relative to the FOV is shown on the right. (**c**) On-axis drug diffusion dynamics along *z* axis. (**d**) Temporal evolution of the on-axis drug signal at a few different z positions. (**e**) Ratio of on-axis signal intensity at 8 h to that at 12 h in (**d**) as a function of axial position.

FOV of the microimaging system is demonstrated in Figure 2a,b. The FOV along the x and y directions is a function of the drive current of the ETL (or equivalently the working distance). The larger the drive current the larger the FOV, as demonstrated in Figure 2a. The linear fit function suggests a FOV(µm)=310+0.49×i(mA) for x and y directions, where i is the drive current of the ETL. The FOV varied between 310 µm and 368 µm when the drive current was changed from 0 to 120 mA which was used in all experiments. This changing lateral FOV leads to a 3D FOV having the shape of a tapered cube (see later in Figure 3b). Figure 2b illustrates that the larger the drive current the smaller the working distance (WD). WD is defined as the axial distance between the focal point and the prism output surface, as demonstrated on the right of Figure 2b. The quadratic fit function suggests $WD\left(\mu m\right)=345.41-1.9844\times i-0.0033\times i^{2}\left(\mathrm{mA}\right)$, resulting in a z direction FOV of 285 µm for the drive current range of 0–120 mA.

Lateral and axial resolutions of the microimaging system were evaluated using the point spread function obtained through 1-µm-diameter fluorescent beads embedded in cured polydimethylsiloxane. The results are demonstrated in Figure 2c,d, respectively. Gaussian fitting suggests a resolution of 2.33 µm, 2.55 µm, and 32.8 µm for x, y, and z direction, respectively. In Figure 2d, the slight shift of the Gaussian curve toward the right is due to the asymmetry of the experimental data around the maximum intensity point. Here, the resolution is defined as full width at half maximum of the fitting curve.

### 2.2. 4D Imaging of Local Drug Delivery

#### 2.2.1. Dynamics of Local Doxorubicin Delivery in Ex Vivo Tissue Phantom

Using the above developed MI-MD system, local delivery of doxorubicin in freshly excised tissue phantom was evaluated. The setup included seven spatially separated reservoirs for drug release (four and three equally separated reservoirs on the left and right side of the opening, respectively), see the left panel of Figure 3a. The axial separation between adjacent reservoirs was 1.1 mm and the axial offset between the two columns was 0.55 mm. The center of the FOV was aligned with the top reservoir on the right side. One image of the drug loaded microdevice is shown in the middle panel of Figure 3a. One image of the sectioning after 12 h of implantation is also shown in the right panel of Figure 3a, where the dark red coloring of the tissue represents the area with drug exposure. Figure 3b displays the 3D images of the drug signal at the time points of 1 h, 3 h, and 12 h, which clearly visualizes the expansion of drug-exposed area over time. See Supplementary Video S1 for visualization of the whole diffusion process. Relative orientation between the 3D image and the GRIN probe is shown by the schematic on the right of Figure 3b. The temporal evolution of the on-axis drug diffusion is examined in Figure 3c, which suggests a rapid increase in overall signal intensity in the early implantation period, followed by a gradual plateau after about 8 h. To achieve the greatest possible optical penetration depth into the tissue, the GRIN probe was moved as close as possible to the capillary tube, and in this case the separation between the lens prism surface and inner capillary wall along the *z* axis was calculated to be around 130 µm. Note that the capillary tube wall thickness was 10 µm, thus it was outside the capillary tube for z positions larger than 140 µm, as indicated by the vertical dashed line in Figure 3c. To show explicitly the drug delivery dynamics reflected in Figure 3c, the signal intensity as a function of time at various z locations is displayed in Figure 3d. In addition, the signal ratio at 8 h to that at 12 h is plotted in Figure 3e. The data in Figure 3d,e indicate quantitatively: (1) the delivery process had completed over 80% after 8 h in comparison to the status at 12 h, and (2) the delivery took longer to reach equilibrium at locations farther away from the drug reservoir (i.e., positions with larger z values).

We note that, due to the absorption of the excitation laser, a minor temperature increase may occur during the 9 min of data acquisition for each 3D image. This temperature rise may result in a slight acceleration of the drug diffusion process, but because the exposure time is relatively short compared to the overall length of drug exposure, we do not expect this to significantly alter the observed drug release kinetics. During our testing, we used low excitation power for image acquisition and blocked the excitation laser between acquisitions to minimize this effect.

#### 2.2.2. Dynamics of Local Doxorubicin Delivery in Murine Tumors

**Figure 4:**
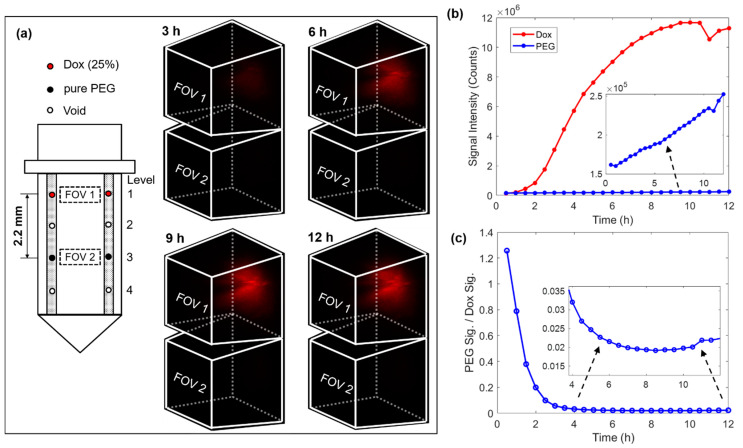
4D imaging of local doxorubicin delivery in murine tumors using the developed MI-MD system. (**a**) Drug loading diagram and the delivery processes captured by the MI-MD system. Dimensions of the FOV and orientation of the imaging probe relative to the FOV are identical to those in Figure 3b. (**b**) Variation of the overall fluorescence signal for both the doxorubicin compound and pure PEG and (**c**) their ratio as a function of time.

The next phase of drug delivery testing was performed in MC38 mouse tumors. For this testing, the microdevice had four reservoirs symmetrically drilled on both sides of the window, see the loading diagram on the left of Figure 4a. A drug loading scheme was chosen in which two reservoirs on the top level were loaded with 25% (*w*/*w*) doxorubicin mixed with polyethylene glycol (PEG) with a molecular weight of 1450 (PEG 1450), and two reservoirs on level 3 were loaded with only PEG 1450 to serve as a control. In this case, the vertical separation between the doxorubicin compound and pure PEG was 2.2 mm. The 3D images on the right of Figure 4a record the drug diffusion patterns at time points of 3 h, 6 h, 9 h, and 12 h. Supplementary Video S2 records the entire diffusion process. There was a well-defined increase in drug signal in FOV 1 (corresponding to the release site of doxorubicin), but there was no detectable drug signal in FOV 2 (corresponding to the control site). Figure 4b summarizes the temporal change of the total integrated fluorescence signal within each FOV. While the fluorescence signal increased steadily on the doxorubicin level, the signal remained very low around the reservoirs on the PEG control level. We observed that the drug signal reaches greater than 90% of its maximum intensity after ~8 h of device implantation. We also observed a decrease in signal after ~10.5 h and determined that it was likely due to the drop in the laser power. Figure 4c plots the ratio between signals from the two levels, which suggests at the stable stage (> 6 h) the signal at the pure PEG level was only around 2% that of the doxorubicin level. It is unclear whether this very low level of signal at the control site indicates crosstalk between these two levels separated by 2.2 mm over the time course of 12 h, or alternatively, very weak autofluorescence from the tumor or the PEG. The diffuse spatial pattern and absence of a clear gradient indicates that it was more likely tissue autofluorescence, but since the microdevice is intended for testing many drugs in parallel, crosstalk between adjacent reservoirs should be minimized. Note that the high ratio in the beginning was due to the low signal level before the drug reached the FOV on the doxorubicin level.

#### 2.2.3. Optical Sectioning of Local Drug Delivery

**Figure 5:**
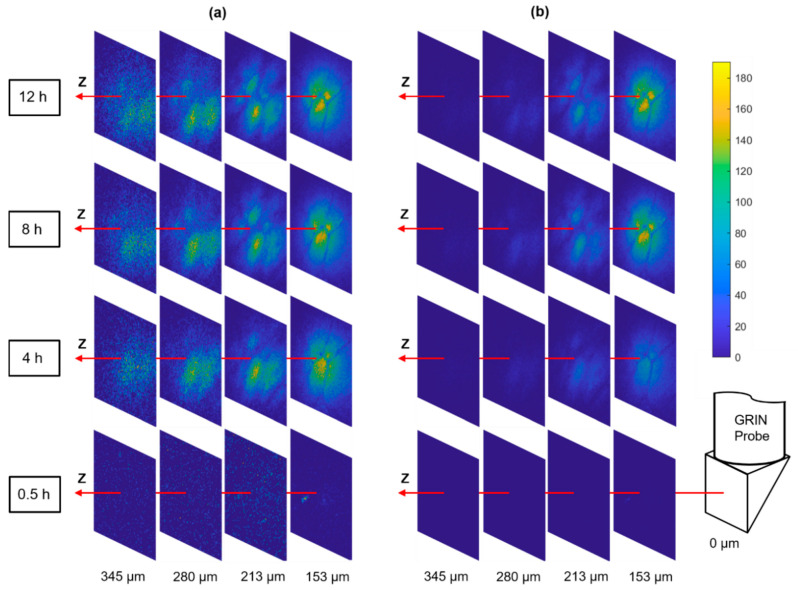
Optical sectioning of the local drug delivery process in tumor. The same images are displayed with (**a**) individual color bars (not shown) for better visualization of the microstructure and (**b**) a common color bar for direct comparison of signal level.

Tumors are known to be very heterogeneous tissues [28], therefore, the drug infiltration after systematic dosing may also be highly nonuniform which has been postulated as an important mechanism of patient drug resistance [6]. The MI-MD system is capable of optically sectioning the drug delivery process in situ, and thereby monitoring the drug penetration through different regions of a heterogeneous tumor. Figure 5a shows a series of distance slices through the optical sectioning for diffusion of the commonly used anti-cancer drug doxorubicin, where the slices use their own color bar to better visualize the microstructure. Figure 5 displays the same images with a common color bar, which allows for a direct comparison of drug signal intensity between slices. A distinct spatial pattern is clearly visualized when the signal levels are above a detection threshold, as shown by the slices at z = 153 µm for the time points of 4 h, 8 h, and 12 h. This pattern was stable over time, which indicates it was originating from the tumor tissue architecture itself rather than a possible nonuniform drug diffusion process.

## 3. Discussion

Investigation of the nonuniform drug distribution is of paramount importance for the thorough evaluation of drug efficacy because several works have highlighted the role of incomplete drug exposure in solid tumors [6]. The 4D MI-MD system is an important upgrade of the system presented by us previously [5]. The extra dimension (scan in z direction) provides a key capability of optically sectioning through the tissue, which enables the direct acquisition of details of the drug distribution. Using the developed MI-MD system, we have successfully demonstrated concurrent release of drug microdoses, and combined it with 4D imaging of local drug delivery in tissue phantoms and mouse tumor models for the first time.

**Figure 6:**
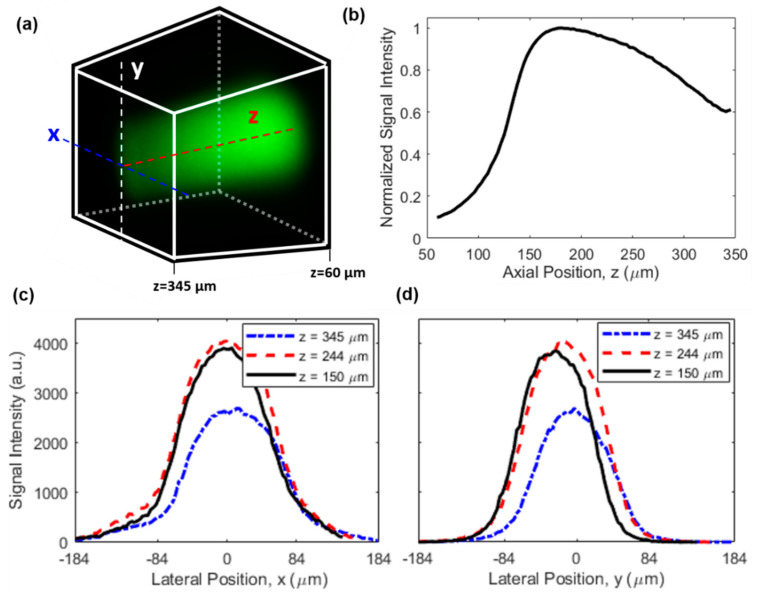
Characterization of the nonuniform signal efficiency. (**a**) A 3D image of a uniform fluorescein solution. Dimensions and orientation of the FOV are identical to those in Figure 3b, see also the coordinate system in the inset of Figure 1a. (**b**) On-axis signal intensity distribution along the z direction. Lateral signal intensity distribution along (**c**) x and (**d**) y direction at different z positions.

For future studies, it will be of high interest to provide absolute quantitation of drug concentration in the tissue to enable a direct comparison with plasma levels and other clinical dosing parameters. One area of further innovation will be to correct for the nonuniform signal efficiency of the microimaging system throughout the 3D FOV, due to the location-dependent excitation and collection efficiencies. This effect leads to a nonuniform image even when the drug concentration is uniform, as revealed by the image in Figure 6a which was acquired from a homogeneous fluorescein aqueous solution. The on-axis signal distribution along *z* axis is shown in Figure 6b, indicating a drop of 40% in signal intensity at the farthest point (around z = 345 µm) relative to the maximum signal intensity (around z = 180 µm) which was in the vicinity of the capillary tube. In the meantime, the signal distribution along lateral directions of x and y is given in Figure 6c,d, respectively. The signal intensity dropped by ~90% when the location was 120 µm away from the center of the FOV (i.e., x = 0) for the x direction, while the signal intensity decreased slightly more rapidly for the y direction. This difference is a result of the asymmetry of the side-viewing prism and the capillary tube in the horizontal (x) and vertical (y) directions. Also, while the signal distribution in the y direction was more symmetric about the peak position, the signal distribution in the x direction exhibited modest asymmetry (e.g., the different curvatures around x = −84 µm and x = 84 µm in Figure 6c), which was likely attributed to slight misalignment between the imaging probe and the capillary tube in the x direction.

**Figure 7:**
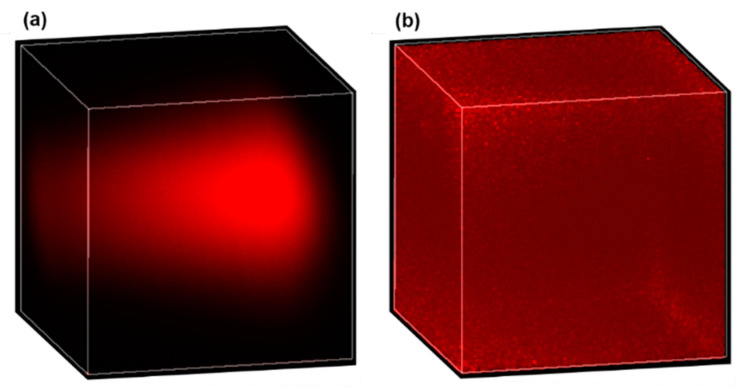
Effective correction for nonuniform signal efficiency by normalization. A 3D image of a uniform 10 µg/mL doxorubicin solution before (**a**) and after (**b**) normalization.

In real tissues, causes of nonuniform signal efficiency are more complicated and may include (1) the varying equivalent numerical aperture (NA), (2) optical losses due to tissue scattering and drug absorption, and (3) aberration originating from propagation in the tissue [29,30]. One possible way to calibrate the nonuniform signal efficiency for quantitative drug measurement is through normalization. To do this, a piece of tissue is first soaked in a drug solution of known concentration for a sufficiently long duration such that uniform drug distribution is established in the tissue. Then, a 3D image of the uniformly drug-soaked tissue is acquired as the calibration signal (defined as $S_{c}$), and described mathematically by $S_{c}=E\left(x,y,z\right)c_{0}$, where E(x,y,z) is the location-dependent signal efficiency and $c_{0}$ is the constant drug concentration. Finally, for real tissues to be imaged, the acquired original 3D image (defined as $S_{o}$, and $S_{o}=E\left(x,y,z\right)c\left(x,y,z\right)$ with c(x,y,z) being the drug concentration distribution) is normalized by the calibration signal, then the location-dependent signal efficiency is removed in the normalized 3D image (defined as $S_{n}$), and mathematically described by $S_{n}=S_{o}/S_{c}=c\left(x,y,z\right)/c_{0}$. Obviously, the signal efficiency E(x,y,z) in the calibration signal and the original image should be the same for this method to work reliably, which requires the calibration signal to be obtained in the same type of tissue. As a preliminary demonstration of the effectiveness of this method, Figure 7 shows the 3D images of doxorubicin solution before and after normalization. Figure 7a is the original 3D image of a 10 µg/mL uniform doxorubicin aqueous solution, while Figure 7b is the normalized 3D image. The calibration signal, which is not shown here, was obtained from a 1 mg/mL doxorubicin solution. The almost uniform distribution in Figure 7b confirms the effectiveness of the normalization method we employed. The larger noise around the corners and edges of the normalized image is because the denominator (i.e., $S_{c}$) is close to zero in those areas, which can be improved by using a higher calibration concentration or a larger pixel dwell time.

Lastly, we want to emphasize that this two-photon MI-MD system is limited to applications where fluorescence imaging is feasible. For settings where non-fluorescent drugs or tissues are used, other label-free imaging techniques, such as Raman imaging, should be applied.

## 4. Materials and Methods

### 4.1. Construction of the Microimaging System

The 3D microimaging system schematically depicted in Figure 1a is a laser scanning microscope equipped with a miniature side-viewing GRIN imaging lens. A laser source (FemtoTrain 1040-3, Spectra-Physics, Milpitas, CA, USA) delivered 1040 nm wavelength pulses with 370 fs pulse width and 350 nJ pulse energy. A high-power optical isolator (ISO-FRDY-05-1030-W, Newport, Irvine, CA, USA) was used directly after the laser output to protect it from destabilizing feedback. The power sent for imaging was adjusted by a variable beam splitter (VA5-PBS253, Thorlabs, Newton, NJ, USA). A remotely controlled optical beam shutter (SHB1T, Thorlabs) was used to block the laser beam between image acquisitions, to avoid excessive illumination of the tissue over the 12 h of testing, thereby reducing the chances of phototoxicity and photobleaching. A 2D galvo system (GVS002, Thorlabs) provided scanning over x and y directions, and an ETL (EL-10-30-TC-VIS-12D, Optotune) along with an offset lens (LC4232, Thorlabs) delivered scanning over the z direction. A dichroic mirror (FF705-Di01, Semrock) was applied to separate the excitation laser and epifluorescence signal. The custom doublet side-viewing GRIN probe (NEM-100-25-100-1040-DM-P9, GRINTECH) has a nominal WD of 250 µm in water and a NA of 0.5 on the object side, and a WD of 1 mm in air and NA of 0.17 on the image side. The large WD on the image side ensured wide z scan range. A 20ꓫ objective lens (PLN 20ꓫ, Olympus) was used to focus the laser beam before the GRIN probe. For the 0–120 mA drive current range of the tunable lens, the objective was tested to have a focus shift of about 1.04 mm which matched closely with the image side WD of the GRIN probe. The laser beam was expanded by four times to match with the input aperture of the objective via a pair of tube lenses with a focus of 50 mm and 200 mm, respectively. A bandpass filter (FF01-605/15-25, Semrock) was used to allow the fluorescence signal to pass through and block the residual excitation wavelength. The filter center wavelength (605 nm) and bandwidth (15 nm) were aligned with the emission spectrum of doxorubicin which peaks around 595 nm [31,32]. The collected epifluorescence was collimated (F950FC-A, Thorlabs) and sent to a PMT (H7421-40, Hamamatsu) for image acquisition via a multimode optical fiber with a core diameter of 1 mm (FP1000ERT, Thorlabs).

### 4.2. Image Acquisition and Displaying

Data acquisition and control of the microimaging system were implemented by a graphical user interface (GUI) developed with LabVIEW. Snake scan was used to acquire the image in the xy plane. Scanning over the z direction was accomplished by tuning the drive current of the ETL with a constant step size. The WD in the z direction was larger with a lower drive current. The control interface of the tunable lens was integrated with the LabVIEW GUI. The image was read out by the PMT operating in the photocounting mode through a terminal block (BNC 2121, National Instruments) connected to a data acquisition device (PCIe 6612, National Instruments). A scan voltage ranging from −2 v to 2 v was sent to each galvo mirror through a multifunction I/O device (National Instruments, PCIe-6351) connected with another terminal block (BNC 2110, National Instruments). There were 100 × 100 × 100 pixels for all the 3D images presented in this work. For the pixel dwell time of 500 µs plus a low time of 10 µs between adjacent pixels, the scan time was around 9 min for each 3D image. For the study in Section 2.2.2, a 3D image was first acquired on the doxorubicin level, then the imaging probe was immediately translated to the pure PEG level for another acquisition. The 3D images were displayed and manipulated using Napari (a multi-dimensional image viewer for Python). All the images were rendered by maximum intensity projection.

### 4.3. Microdevice Fabrication and Drug Loading

MI-MD microdevices were fabricated by a CNC 5-axis micromachining station (MDA, TN5-V8-TC8) using medical-grade Delrin. The microdevices had overall shape and dimensions shown in Figure 1b. The flange was designed to fix the microdevice on the cap of a custom-made tissue holder and facilitate the implantation into tissue. A 90° side-viewing window was machined on one side of the microdevice (see also the cross-sectional view in the right image of Figure 3a), and micro reservoirs of 0.2-mmdiameter and 0.2-mm depth were drilled on both sides of the opening. The vertical separation between adjacent reservoirs was 1.1 mm. A capillary tube (Quartz 20-QZ, Charles Supper) with nominal outer diameter of 2 mm and wall thickness of 10 µm was inserted into the microdevice center hole to protect the imaging probe from contamination. No index matching liquid between the capillary tube and the imaging probe was used. The micro reservoirs were loaded with doxorubicin powder (Selleck Chem., Houston, TX, USA) mixed with 1450 g/mole molecular weight PEG, as described in [1]. The doxorubicin concentration was 25% by weight. More details about the microdevice manufacturing, drug formulation, and drug loading can be found in our previous work [1,5].

### 4.4. Preparation of Tissues

Fresh chicken breast tissues were used as ex vivo tissue phantoms for studies in Section 2.2.1. These were cut into small pieces and put in the tissue holder, which had a cap on which the microdevice was fixed. The tissue was capped during testing to keep it from drying out over the 12 h of testing.

Murine subcutaneous colon adenocarcinoma (MC38) tumors were used in Section 2.2.2. MC38 cell lines (ENH204-FP, Kerafast) for the murine tumors were established by standard protocols, and they were cultured in complete DMEM with 10% FBS and 5% penicillin-streptomycin. The cells were tested and confirmed negative for mycoplasma before use. Tumors on BL6 mice (C57BL/6J, Jackson) were initiated by injecting approximately 200 µL of the cell suspension (10 million cells/mL) into the flank region on both sides under 1–3% isoflurane anesthesia. Tumors grew for about three weeks and reached up to 1–1.5 cm maximal diameter. The tumors were flash frozen after being harvested and stored at −80 °C prior to use. All the procedures followed the institutional animal care and use committee protocol which was approved.

## 5. Conclusions

A MI-MD system with optical sectioning capabilities has been constructed, which enables simultaneous release of multiple drug microdoses into tumors and real-time 3D imaging of spatial drug release. The microimaging system is a two-photon imaging and GRIN lens-based 3D micro-endoscope. Using the MI-MD system, dynamics of doxorubicin released locally into tissue phantoms and murine tumors has been successfully acquired with 3D spatial resolution and across time. In addition, optical sectioning of nonuniform drug distribution in a tumor with highly heterogeneous structure has been demonstrated successfully. A quantitative imaging method that corrects for space-dependent anisotropy in signal efficiency by normalization was also discussed. Future advances of this research may include the simultaneous monitoring of drug release and tissue response using a multi-color MI-MD system, which may find promising applications in the in vivo testing of drug efficacy.

## Data Availability

The data presented in this study are available upon request from the corresponding author.

## Supplementary Materials

The following are available online at https://www.mdpi.com/article/10.3390/ijms222111752/s1.
